# *Lactobacillus plantarum* ZJUIDS14 alleviates non-alcoholic fatty liver disease in mice in association with modulation in the gut microbiota

**DOI:** 10.3389/fnut.2022.1071284

**Published:** 2023-01-09

**Authors:** Feiwei Cao, Qinchao Ding, Hui Zhuge, Shanglei Lai, Kaixin Chang, Chunyan Le, Guorong Yang, Teresa G. Valencak, Songtao Li, Daxi Ren

**Affiliations:** ^1^College of Animal Sciences, Institute of Dairy Science, Zhejiang University, Hangzhou, China; ^2^School of Public Health, Zhejiang Chinese Medical University, Hangzhou, China; ^3^School of Life Sciences, Zhejiang Chinese Medical University, Hangzhou, China

**Keywords:** *Lactobacillus plantarum* ZJUIDS14, non-alcoholic fatty liver disease, hepatic steatosis, mitochondrial function, intestinal microbiota

## Abstract

This present study was designed to explore the protective role of *Lactobacillus plantarum* ZJUIDS14 against Non-alcoholic Fatty Liver Disease (NAFLD) in a high-fat-diet (HFD)-induced C57BL/6 mice model. The probiotic (10^9^ CFU/every other day) was administered by oral gavage for 12 weeks. We found that *L. plantarum* ZJUIDS14 intervention significantly alleviated HFD related hepatic steatosis, liver damage, insulin resistance, and increased hepatic expression of peroxisome proliferator activated receptor α (PPAR-α) while stimulating the activation of AMP-activated protein kinase (AMPK). Furthermore, *L. plantarum* ZJUIDS14 improved mitochondrial function as reflected by an increase in dynamin related protein 1 (DRP1) and a decrease of proteins associated with oxidative phosphorylation (OXPHOS) after the treatment. Additionally, mice from the *L. plantarum* ZJUIDS14 group had a restored intestinal flora and homeostasis involving *Coprostanoligenes group*, *Ruminococcaceae UCG*-*014*, *Allobaculum*, *Ruminiclostridium 1*, and *Roseburia*. Meanwhile, these five genera exhibited a significant (negative or positive) association with ileum inflammation mRNA levels and SCFA contents, by Spearman’s correlation analysis. In general, our data demonstrated that *L. plantarum* ZJUIDS14 mitigates hepatic steatosis and liver damage induced by HFD. Specifically, they strengthened the integrity of the intestinal barrier, regulated gut microbiota, and improved mitochondrial function. Our data provide an experimental basis for *L. plantarum* ZJUIDS14 as a promising candidate to prevent NAFLD.

## 1. Introduction

Non-alcoholic fatty liver disease (NAFLD) has become a rapidly increasing disease in incidence over the past decades (1, 2), which prevalence increased from 25.5% in or before 2005 to 37.8% in 2016 or later and is considered the precursor of steatohepatitis (NASH), liver fibrosis, cirrhosis, and even hepatocellular carcinoma (HCC) (3). The pathogenesis of NAFLD is generally described as being caused by multiple factors (4). NAFLD starts from mild steatosis, caused by excessive hepatic fat deposition, which progresses to NASH under multiple influences including oxidative stress and inflammation (5). To date, there still is no effective treatment for NAFLD (6). Therefore, suitable dietary supplements that can mitigate or even prevent NAFLD without toxic side-effects are critically sought after.

Several studies have reported that gut microbiota influence the pathophysiology of NAFLD, along the gut-liver axis (7). Gut microbiota and their metabolites, such as short chain fatty acids (SCFAs), play important roles. For example, administration of SCFAs can decrease the lipid accumulation in the liver of animal models of obesity and T2DM, via increasing hepatic AMPK phosphorylation and expression of PPARα, and further stimulating FFA oxidation (8–10).

Probiotics, living microorganisms with beneficial effects on the host, have previously received more and more attention (11). There are several studies on the effects of different probiotics on improving NAFLD. VSL#3, mixed with 8 probiotic strains (*Lactobacillus plantarum*, *bifidobacteria* [*Bifidobacterium breve*, *Bifidobacterium infantis*, *Bifidobacterium longum*], *Lactobacillus acidophilus*, *Lactobacillus paracasei*, *Streptococcus thermophilus*, and *Lactobacillus delbrueckii subsp*. *bulgaricus*), was observed to have a protective role in NAFLD, both in humans and in mice (12–14). In a double-blind RCT experiment, VSL#3 was administered to obese children for 4 months, and children in the VSL#3 group had lower BMIs and higher GLP-1 levels compared to children in the placebo group (15). Yu et al. (16) *treated w*estern diet-induced NAFLD mice with *Lactobacillus lactis* and *Pediococcus pentosaceus* in the drinking water at a concentration of 10^9^ CFU/g. After 8 weeks, mice from both probiotic supplementation groups maintained key metabolic features such as bile acids (BAs), SCFAs, tryptophan metabolites, and better NAFLD activity scores, cytokines, biochemical markers, and gut-tight junctions. Chen et al. (17) treated HFD-induced mice with 10^9^ CFU/g *L. plantarum* FZU3013 for 8 weeks. The results showed that *L. plantarum* FZU3013 prevented NAFLD and hyperlipidemia by modulating specific intestinal microbial phylotypes and increasing the expression levels of genes relating to lipid metabolism, including the bile salt export pump (BSEP) and cholesterol 7α-hydroxylase (CYP7A1). However, the mechanism by which *L. plantarum* prevents NAFLD remains unclear.

In our previous study, *L. plantarum* ZJUIDS14, isolated from feces from human babies, reportedly improved chronic alcohol-induced liver disease in mice (18, 19). *L. plantarum* ZJUIDS14 was resistant to stomach acids and bile salts, and showed no signs of antibiotic resistance. However, its effects and mechanism on preventing NAFLD are still unknown. Therefore, the objective of this study was to investigate the preventive effects of *L. plantarum* ZJUIDS14 in HFD-fed mice, and thus to gain more functional insights into the role of lactic acid bacteria (LAB) for preventing NAFLD.

## 2. Materials and methods

### 2.1. Probiotic

*Lactobacillus plantarum* ZJUIDS14 were isolated from feces sampled from breastfed babies (6-month old) in Hangzhou, Zhejiang Province, China. This strain was lyophilized (WECARE-BIO Biotechnology Co. Ltd., Jiangsu, China) and stored at −80°C until use.

### 2.2. Animal experiment design

Eight-week-old male C57BL/6 mice were purchased from B&K Experimental Animal Corporation Ltd. (License No. SCXK(Hu) 2013-0016). C57BL/6 mice were housed in cages at 23 ± 2°C and 55 ± 5% relative humidity under a 12 h light/dark cycle. After 1 week of acclimatization, mice were frandomly assigned to three experimental groups: normal-food-diet (NFD, *n* = 8), high-fat-diet (HFD, *n* = 8) and high-fat-diet with ZJUIDS14 group (HFD + ZJUIDS14, *n* = 8). The NFD diet (10% fat, D12450B) and HFD diet (45% fat, D12451) were purchased from Research Diets, Inc. (New Brunswick, NJ, USA). *L.* p*lantarum* ZJUIDS14 were administered at a dosage of 10^9^ CFU/every other day for 12 weeks, before mice were euthanized with pentobarbital solution after 12 h of fasting (Supplementary Figure 1). Then, livers, small intestine, colon contents and blood samples were collected for further analyses.

All the animal procedures were approved by the Institutional Animal Care and Use Committee of Zhejiang Chinese Medical University and the animals were maintained according to the guidelines of the Animal Experimental Center of Zhejiang Chinese Medical University (approval number: IACUC-20210621-05).

### 2.3. Oral glucose tolerance test and insulin tolerance test

For OGTT, mice were injected glucose at a dosage of 2.5 g/kg body weight after 12 h fasting. Blood samples were taken at regular time points (0, 30, 60, 120 min) and glucose concentrations were measured using a glucometer (Sinocare).

For ITT, mice were fasted for 4 h and each mouse was injected insulin (Solarbio, Beijing, China) at 0.75 U/kg body weight. Blood glucose was measured at 0, 30, 60 and 120 min after the insulin injection.

### 2.4. Histological analysis

The liver tissues were fixed in 4% paraformaldehyde for 24 h. Subsamples of hepatic tissues were embedded in paraffin, tissues were cut into 4 μm sections, stained with hematoxylin and eosin (H&E) and analyzed by light microscopy. NAFLD Activity Scores (NAS) were obtained in a blinded fashion for mouse livers and cross-validated by a pathologist. NAS was determined by the degree of steatosis, inflammation, and ballooning.

### 2.5. Biochemical analyses

Plasma was obtained by centrifugation with 2,000 rpm at 4°C for 15 min, and then stored at −80°C until analysis. Plasma levels of alanine transaminase (ALT) and aspartate transaminase (AST) were determined by AST and ALT determination kit (Nanjing Jiancheng Bio Co., Nanjing, China). Total cholesterol (TC), low-density cholesterol (LDL) and high-density cholesterol (HDL) in plasma were detected by TC, LDL and HDL determination kit (Applygen, Beijing, China). The levels of TG in the liver tissues were determined using a liver TG determination kit (Applygen, Beijing, China). Plasma endotoxin levels were determined using a limulus amebocyte lysate kit (Bioendo, Xiamen, China). In addition, the contents of MDA and enzyme activities of T-SOD were determined by MDA and T-SOD determination kit (Nanjing Jiancheng Bio Co., Nanjing, China).

### 2.6. Quantitative real-time polymerase chain reaction

Total RNA was isolated from the liver or ileum using Trizol reagent (Invitrogen, Carlsbad, USA), and then transcribed into cDNA by reverse transcription reagent kits (Monad, Wuhan, China) according to the manufacturer’s protocol. The primer sequences (Sangon Biotech, Shanghai, China) are given in Table 1. The data were normalized to 18s rRNA mRNA levels and presented as fold changes, setting the value of controls as 1.

**TABLE 1:** Primer sequence for qRT-PCR.

Gene		Primer sequence (5′–3′)
*srebp1c*	Forward Reverse	CCACCTCGTGAACGAAGATT ACCACCACGACCTCGAATAG
*cd36*	Forward Reverse	ATGGGCTGTGATCGGAACTG GTCTTCCCAATAAGCATGTCTCC
*ppar-α*	Forward Reverse	TGCCTTCCCTGTGAACTGAC TGGGGAGAGAGGACAGATGG
*dgat2*	Forward Reverse	AGTGGCAATGCTATCATCATCGT TCTTCTGGACCCATCGGCCCCAGGA
*fatp2*	Forward Reverse	TCCTCCAAGATGTGCGGTACT TAGGTGAGCGTCTCGTCTCG
*fatp5*	Forward Reverse	CTACGCTGGCTGCATATAGATG CCACAAAGGTCTCTGGAGGAT
*fabp2*	Forward Reverse	CTGGCATGTGAGGCGGTTA GCCGTCGAATGCCATGATCT
*cramp*	Forward Reverse	TCTCTACCGTCTCCTGGACCTG CCACATACAGTCTCCTTCACTCG
*IL-1β*	Forward Reverse	GAAATGCCACCTTTTGACAGTG TGGATGCTCTCATCAGGACAG
*TNF-α*	Forward Reverse	CCCTCACACTCAGATCATCTTC GTTGGTTGTCTTTGAGATCCAT
*18S*	Forward Reverse	AGGTCTGTGATGCCCTTAGA GAATGGGGTTCAACGGGTTA

### 2.7. Western blotting

Protein levels from the liver and intestine were assessed by western blotting. In brief, liver tissue or cells were lysed with RIPA buffer (Boster Biological Technology, Wuhan, China) supplemented with protease and phosphatase inhibitors (Sigma-Aldrich, St. Louis, MO, USA). The protein samples were loaded into SDS-PAGE gels and transferred to PVDF membranes. The membranes were blocked with 5% non-fat milk in TBST and then were allowed to react with primary antibodies (Supplementary Table 1) at 4°C for 12 h. After washing with TBST, the membranes were incubated with horseradish peroxidase-conjugated secondary antibody for 1 h at room temperature. Finally, the immunoreactivity of protein expression was visualized with an electrogenerated chemiluminescence kit (Vazyme, Nanjing, China). The immunoblots were quantified by measuring the density of each band with Image-J.

### 2.8. 16s rRNA gene sequencing

The collected colonic content samples were sent to Mingke Biotechnology (Hangzhou) Co., Ltd. for total DNA isolation and 16s rRNA high-throughput sequencing technology. The 16s rRNA was amplified at the V3–V4 region. The amplicons were purified with a QIA quick PCR purification kit (Qiagen, Hilden, Germany), the sequencing was conducted by an Illumina MiSeq platform (PE250, San Diego). Raw sequences were subjected to a quality-control process via UPARSE. Analyses of *amplicon sequence variants* (ASVs) were performed using the platform QIIME2. The 16S rRNA gene sequences were aligned against the SILVA v132.

### 2.9. Determination of SCFA levels in fecal samples

The fecal samples were diluted three-fold with ultrapure water and vortexed for 5 min. Next, the suspension was allowed to rest for 5 min and then centrifuged at 4°C, 5,000 × *g* for 20 min. One milliliter of supernatant was mixed with 20 μL chromatogram grade phosphoric acid (Shanghai Aladdin Biochemical Technology Co., Ltd.), and the mixture was injected into a chromatographic vial (WondaVial, Shimadzu, Corp., Kyoto, Japan) through a 0.45 μm membrane filter for gas chromatography. The SCFA content of the mixture was detected by a gas chromatograph (Shimadzu, Corp., Kyoto, Japan), as previously described (20).

### 2.10. Statistical analyses

The analyses were performed using GraphPad Prism (GraphPad Software 8.0.1). Data are expressed as the mean ± standard deviation (SD). Results were analyzed using a two-way analysis of variance (ANOVA). In all tests, *P* values of less than 0.05 were considered statistically significant. Correlation analysis was conducted between different filtered fecal bacteria and NAFLD parameters using Spearman rank correlation and shown by heatmaps reflecting mean values of Spearman’s. The permutational multivariate analysis of variance (PERMANOVA) was used to compare beta-diversity between groups.

## 3. Results

### 3.1. *Lactobacillus plantarum* ZJUIDS14 administration ameliorates hepatic steatosis and liver damage in HFD-fed mice

**FIGURE 1:**
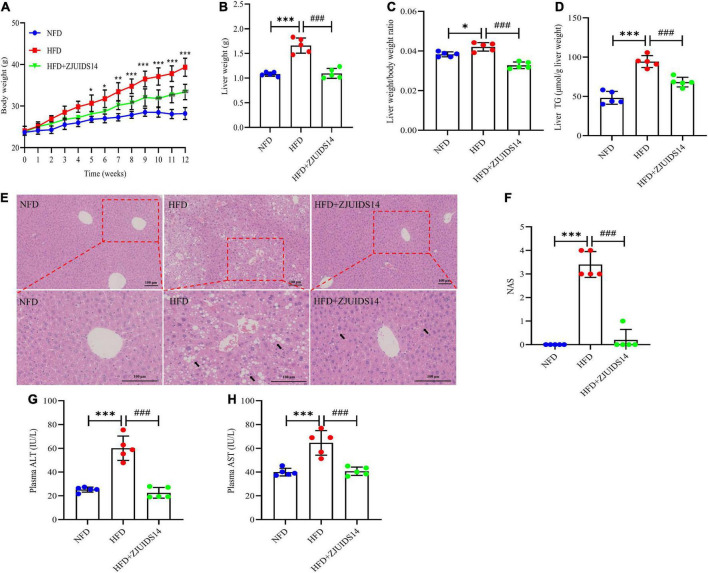
*Lactobacillus plantarum* ZJUIDS14 administration ameliorates hepatic steatosis and liver damage in HFD-fed mice. **(A)** Body weights; **(B)** liver weights; **(C)** liver weight/body weight ratio; **(D)** liver TG; **(E)** liver H&E staining, magnification. **(F)** NAFLD activity score (NAS); **(G)** plasma ALT; **(H)** plasma AST. *P < 0.05, ^**^P < 0.01, ^***^P < 0.001 *vs* NFD group; *^###^P* < 0.001 *vs* HFD group.

Mice fed HFD for 12 weeks had significantly increased body weights, liver weights, and liver/body weight ratios (%) compared with the NFD group (Figures 1A–C), which were significantly reduced in ZJUIDS14-treated mice. Diet-induced steatosis was prevented by *L. plantarum* ZJUIDS14 treatment, as indicated by lower triglyceride accumulation in the livers of *L. plantarum* ZJUIDS14 treated mice (Figure 1D). H&E staining showed that the livers contained different sizes of lipid droplets in the HFD group, while the livers showed decreased droplet sizes and an intact structure in the *L. plantarum* ZJUIDS14 group (Figure 1E). NAFLD activity score (NAS) also confirmed that supplementation with *L. plantarum* ZJUIDS14 inhibited the progression of NAFLD in HFD fed mice (Figure 1F). Both ALT and AST were significantly enhanced by HFD while decreased by supplementation with *L. plantarum* ZJUIDS14 (Figures 1G, H). Our data clearly show that *L. plantarum* ZJUIDS14 could effectively reduce HFD-induced lipid accumulation and liver damage.

### 3.2. *Lactobacillus plantarum* ZJUIDS14 administration reduces insulin resistance and dyslipidemia in HFD mice

**FIGURE 2:**
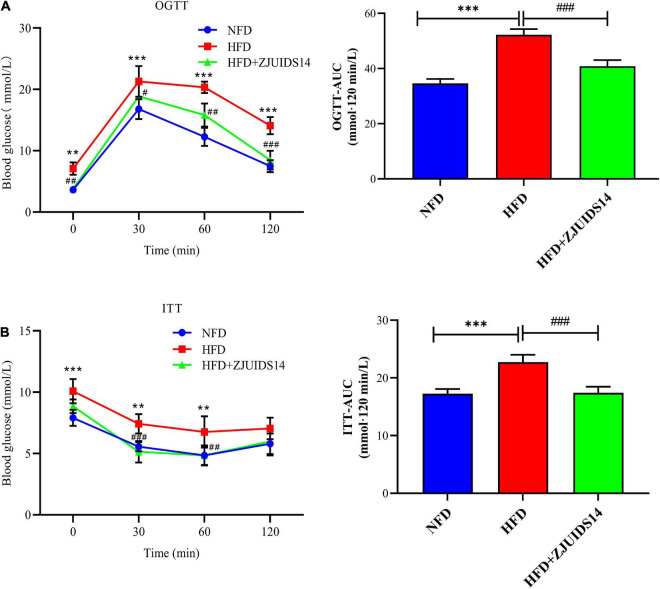
*Lactobacillus plantarum* ZJUIDS14 administration reduces insulin resistance in HFD fed mice. **(A)** Oral glucose tolerance tests (OGTT) and **(B)** insulin tolerance tests (ITT). ^**^P < 0.01, ^***^P < 0.001 *vs* NFD group; *^#^P* < 0.05, *^##^P* < 0.01, *^###^P* < 0.001 *vs* HFD group.

**FIGURE 3:**
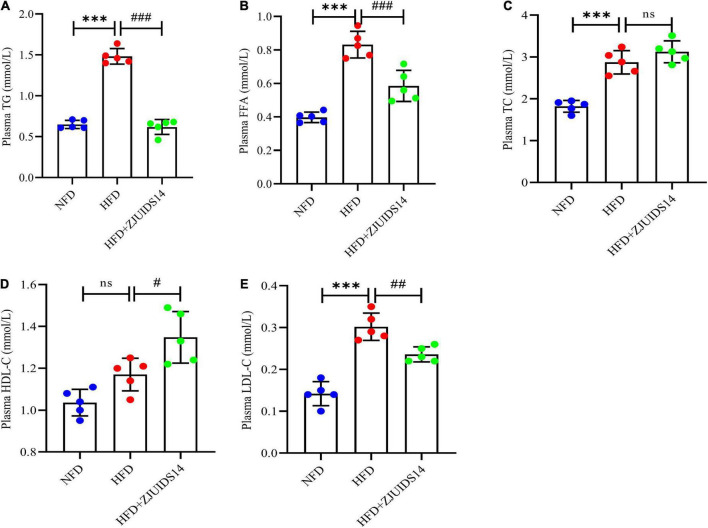
*Lactobacillus plantarum* ZJUIDS14 administration reduced dyslipidemia in HFD fed mice. **(A)** Plasma TG; **(B)** plasma FFA; **(C)** plasma TC; **(D)** plasma HDL-C; **(E)** plasma LDL-C. ^***^P < 0.001 *vs* NFD group; *^#^P* < 0.05, *^##^P* < 0.01, *^###^P* < 0.001 *vs* HFD group. ns, not significant.

OGTT revealed that *L. plantarum* ZJUIDS14 significantly improved glucose clearance (Figure 2A). Improved insulin sensitivity was also observed in the HFD + ZJUIDS14 group, as taken from ITT (Figure 2B). Besides, plasma TG, FFA, TC and LDL-C levels were significantly increased in response to HFD compared to the control group (Figures 3A–C, E). However, *L. plantarum* ZJUIDS14 treatment significantly prevented increasing TG, FFA, and LDL-C levels (*P* < 0.01). In addition, HDL-C levels were elevated under treatment with *L. plantarum* ZJUIDS14 (Figure 3D).

### 3.3. *Lactobacillus plantarum* ZJUIDS14 administration inhibits hepatic fat accumulation in HFD mice

**FIGURE 4:**
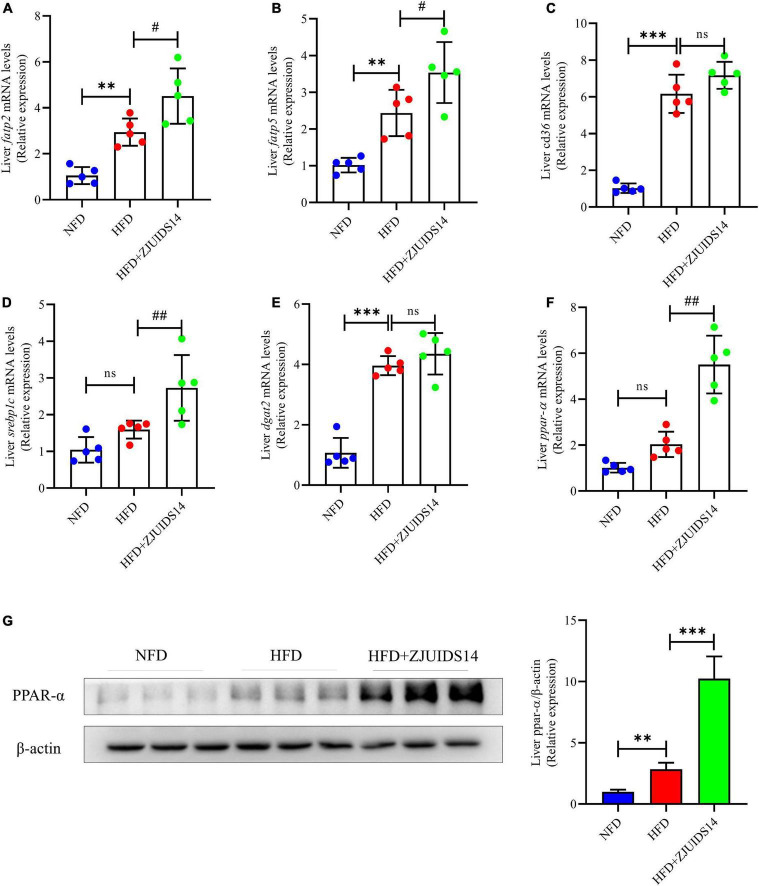
*Lactobacillus plantarum* ZJUIDS14 administration inhibits hepatic steatosis in HFD fed mice. Hepatic mRNA expressions of **(A)** *fatp2*, **(B)** *fatp5*, **(C)** *cd36*, **(D)** *srebp1c*, **(E)** *dgat2*, **(F)** *ppar-α*, **(G)** protein expression of PPAR-α. ^**^P < 0.01, ^***^P < 0.001 *vs* NFD group; *^#^P* < 0.05, *^##^P* < 0.01 *vs* HFD group. ns, not significant.

To further explore the mechanism underlying the protective role of *L. plantarum* ZJUIDS14 against hepatic steatosis, gene expression of lipogenesis genes in the liver was observed by RT-qPCR. Genes regulating fatty acid uptake, including cluster of differentiation 36 (*cd36*), fatty acid transport protein 2 (*fatp2*) and fatty acid transport protein 5 (*fatp5*), were higher in HFD fed mice when compared with the NFD mice (*P* < 0.05). In addition, *L. plantarum* ZJUIDS14 further increased *fatp2* and *fatp5* (Figures 4A, B). However, *cd36* was not affected by *L. plantarum* ZJUIDS14 treatment (Figure 4C). Hepatic expression levels of *srebp1c* and diacylglycerol O-acyltransferase 2 (*dgat2*) were higher in the HFD group than the NFD group (Figures 4D, E). There was no significant difference in expression levels of *ppar-α* (Figure 4F). The genes *ppar-α*, *srebp1c, fatp2*, and *fatp5* were increased in the livers of mice treated with *L. plantarum* ZJUIDS14. Consistent with the RT-qPCR results, the protein expression of was increased by *L. plantarum* ZJUIDS14 supplementation (Figure 4G). The results showed that *L. plantarum* ZJUIDS14 could promote fat oxidation and prevent TG accumulation.

### 3.4. *Lactobacillus plantarum* ZJUIDS14 administration improves mitochondrial function in HFD fed mice

**FIGURE 5:**
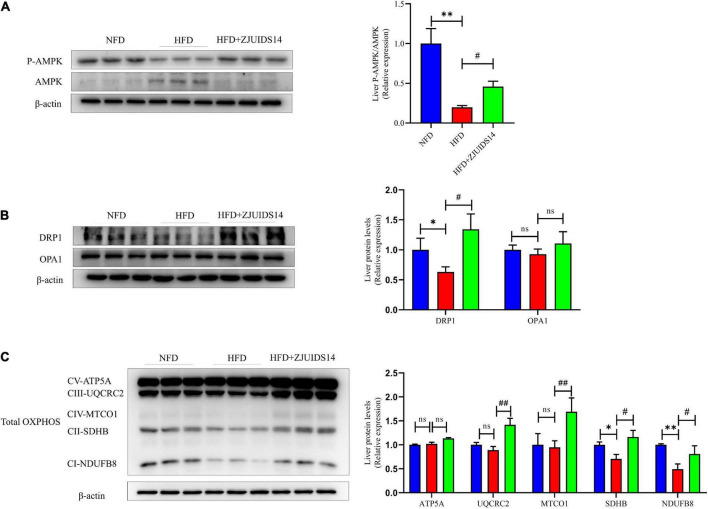
*Lactobacillus plantarum* ZJUIDS14 administration improves mitochondrial function in HFD fed mice. Protein levels of **(A)** P-AMPK and AMPK, **(B)** DRP1 and OPA1, **(C)** OXPHOS. *P < 0.05, ^**^P < 0.01 *vs* NFD group; *^#^P* < 0.05, *^##^P* < 0.01 *vs* HFD group. ns, not significant.

AMP-activated protein kinase (AMPK) plays an important role in stimulating hepatic fatty acid oxidation and as such is considered as a target to inhibit NAFLD (21). We observed that HFD feeding decreased p-AMPK levels and increased AMPK levels, indicating that HFD might have reduced AMPK activation in NAFLD livers (Figure 5A). Remarkably, after treatment with *L. plantarum* ZJUIDS14 the levels of p-AMPK and AMPK were restored, indicating enhancement in fatty acid oxidation. Relating to the mitochondrial fission process, the expression levels of dynamin related protein 1 (DRP1) and optic atrophy 1 (OPA1) were determined. Our results demonstrated that HFD significantly reduced DRP1, which was increased by *L. plantarum* ZJUIDS14 administration (Figure 5B). There were no significant changes of OPA1 between the three feeding groups (Figure 5B). Together, our data suggest that *L. plantarum* ZJUIDS14 restored the impaired fission process in HFD fed mice. Five mitochondrial proteins (ATP5A, UQCRC2, MTCO1, SDHB, and NDUFB8) were analyzed and there was no significant change in ATP5A protein expression between the different groups (Figure 5C). UQCRC2, MTCO1, SDHB, and NDUFB8 protein expression levels were significantly decreased (*P* < 0.05) in the HFD group compared to the NFD group and significantly higher (*P* < 0.05) in the *L. plantarum* ZJUIDS14 group compared to HFD feeding without the probiotic (Figure 5C). Oxidative stress affects the etiology of NAFLD by its “second hit” action. To evaluate the effects of *L. plantarum* ZJUIDS14 on oxidative stress, the levels of liver MDA and liver T-SOD were tested. The results show that HFD indeed increased MDA and decreased T-SOD (Supplementary Figures 3A, B). However, these changes were inhibited by *L. plantarum* ZJUIDS14 administration significantly. To explore the functional mechanism of antioxidant-effects, nuclear factor erythroid 2-related factor 2 (NRF2) signaling-associated proteins Nrf2, heme oxygenase-1 (HO-1), and mitochondria-related protein HO-2 were determined by Western-blotting. HFD decreased Nrf2 and HO-1 protein levels, and increased HO-2 levels (Supplementary Figure 3C). After *L. plantarum* ZJUIDS14 supplementation, HO-2 levels were further increased, whereas levels of Nrf2 and HO-1 remained stable. These results suggested that the probiotic *L. plantarum* ZJUIDS14 exerted antioxidant effects via the mitochondria-dependent pathway but not the Nrf2 signaling pathway.

### 3.5. *Lactobacillus plantarum* ZJUIDS14 administration ameliorates ileum lipid metabolism and inflammation in HFD fed mice

**FIGURE 6:**
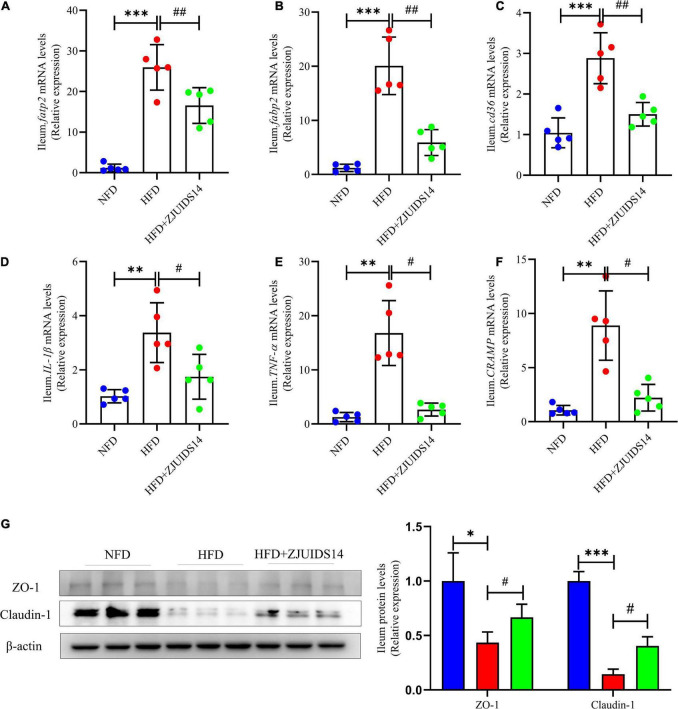
*Lactobacillus plantarum* ZJUIDS14 administration mitigates ileum lipid metabolism and inflammation in HFD fed mice. Relative mRNA expression levels of **(A)** *fatp2*; **(B)** *fabp2*; **(C)** *cd36*; **(D)** *IL-1β*; **(E)** *TNF-α*; and **(F)** *cramp* in ileum, protein levels of **(G)** ZO-1 and Clautin-1 in ileum. *P < 0.05, ^**^P < 0.01, ^***^P < 0.001 *vs* NFD group; *^#^P* < 0.05, *^##^P* < 0.01 *vs* HFD group.

Expression of lipogenic genes was increased in ileum tissues of HFD fed mice, and was decreased after *L. plantarum* ZJUIDS14 treatment, including *fatp2*, *fatty-acid-binding protein 2 (fabp2)*, and *cd36* (Figures 6A–C). Also, *L. plantarum* ZJUIDS14 supplementation produced statistically significant changes in mRNA levels of interleukin-1 beta (*IL-1β)* and tumor necrosis factor-alpha (*TNF-α*; Figures 6D, E), compared with HFD mice. However, mRNA expression of Cathelicidin-related antimicrobial peptide (*cramp*) in ileum was higher in HFD compared to NFD group, but it was decreased in the *L. plantarum* ZJUIDS14 group (Figure 6F). In addition, ZJUIDS14 significantly inhibited increasing levels of tight junction proteins (Claudin-1 and ZO-1) in ileum induced by HFD (Figure 6G). Thus, the NAFLD-counteracting effects of *L. plantarum* ZJUIDS14 in HFD-fed mice may be caused by direct modulation of inflammation in the gastrointestinal tract.

### 3.6. *Lactobacillus plantarum* ZJUIDS14 administration rescues gut microbiota dysbiosis in HFD mice

**FIGURE 7:**
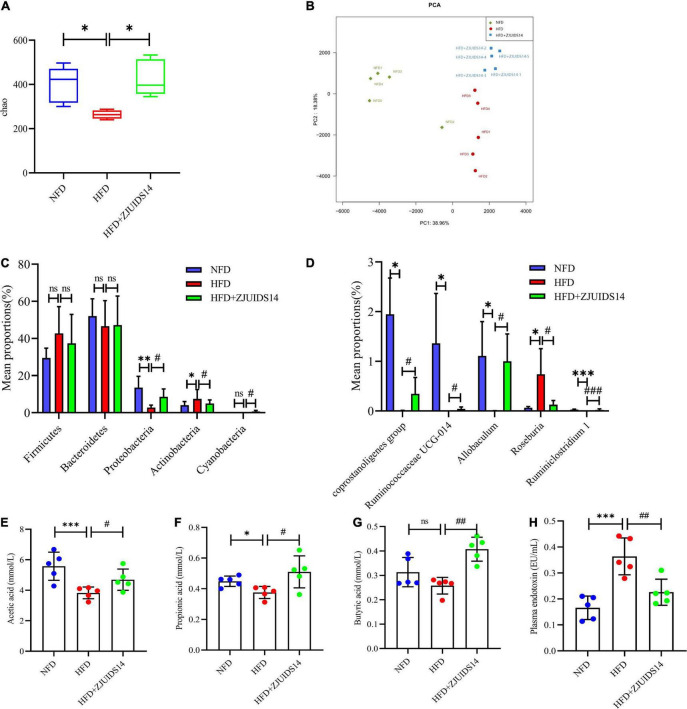
*Lactobacillus plantarum* ZJUIDS14 administration prevents gut microbiota dysbiosis in HFD fed mice. **(A)** Alpha diversity using Chao richness. **(B)** Principal coordinate analysis. Different fecal microbiota profiles shown at phylum **(C)** and genus **(D)** level. Intestinal contents of **(E)** acetic acid, **(F)** propionic acid, **(G)** butyric acid. **(H)** Plasma endotoxin. *P < 0.05, ^**^P < 0.01, ^***^P < 0.001 *vs* NFD group; *^#^P* < 0.05, *^##^P* < 0.01, *^###^P* < 0.001 *vs* HFD group. ns, not significant.

To better explore the changes in microbial ecology in response to HFD and *L. plantarum* ZJUIDS14 administration, the composition of microbiota was broadly identified via 16S rRNA amplicon pyrosequencing and SCFAs levels from ileum contents of all groups. Species richness (Chao indexes), measured by numbers of observed OTUs, were compared among three groups. The microbiome in mice fed HFD by trend had reduced microbial richness, with a decreased chao index compared to the control group (Figure 7A). However, richness was maintained in individuals provided with *L. plantarum* ZJUIDS14, that had significantly increased chao indices compared to the HFD group (*P* < 0.01). As for beta diversity, PERMANOVA analysis revealed significant differences in the gut microbial community between the HFD and NFD samples (*R*^2^:0.75, *P* < 0.01) and between the HFD + ZJUIDS14 and HFD samples (*R*^2^:0.53, *P* < 0.01; Figure 7B). At the phylum level (Figure 7C), *Bacteroidetes* and *Firmicutes* had the highest abundances and were the dominant bacteria in the three groups. However, there was no significant difference in *Bacteroidetes* and *Firmicutes* richness among the three groups. Compared with the NFD group, HFD mice exhibited a decreased percentage of *Proteobacteria* and an increased percentage of *Actinobacteria* (*P* < 0.05). *L. plantarum* ZJUIDS14 treatment mitigated these changes as well as the decreased level of *Cyanobacteria* (*P* < 0.05). At the genus level (Figure 7D), the relative abundances of *Coprostanoligenes* group, *Ruminococcaceae UCG-014, Allobaculum*, and *Ruminiclostridium 1* were significantly reduced with that of *Roseburia* being increased by HFD group, compared to NFD group (*P* < 0.05). However, these changes were absent following *L. plantarum* ZJUIDS14 treatment (*P* < 0.05). As shown in Figures 7E–G, HFD mice had decreased levels of acetic acid, propionic acid, and butyric acid, compared to NFD mice (*P* < 0.05). SCFA production was elevated when mice were supplemented with *L. plantarum* ZJUIDS14 (*P* < 0.05). Moreover, HFD caused significant increase in plasma endotoxin (Figure 7H) compared with NFD mice (*P* < 0.05), while supplementation of ZJUIDS14 lowered plasma endotoxin (*P* < 0.05). Collectively, our data demonstrate that HFD feeding leads to dysbiosis in cecal microbiota, which was ameliorated by the probiotic *L. plantarum* ZJUIDS14.

### 3.7. Correlation between the gut microbiota and NAFLD related parameters

**FIGURE 8:**
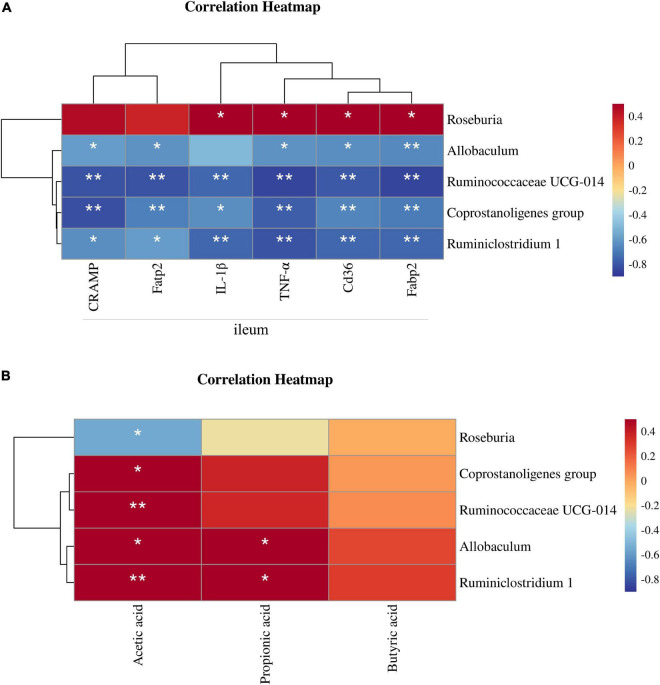
Correlation between gut microbiota and NAFLD parameters. **(A)** Heatmap of the Spearman’s correlations between the bacterial genera and the levels of ileum mRNAs. **(B)** Heatmap of the Spearman’s correlations between the bacterial genera and the SCFA contents. The Spearman correlation coefficients are shown in different colors. *P < 0.05, **P < 0.01.

The Spearman correlation coefficient was calculated among the NFD, HFD, and HFD + ZJUIDS14 groups of mice to show the effects of NAFLD related parameters on gut specific bacterial genera by hierarchical clustering. According to the Spearman correlation heatmap analysis at the genus level, *Roseburia* showed a significant positive relationship with expression of IL-1β, TNF-α, cd36, and fabp2 in ileum of mice. In contrast, *Ruminiclostridium 1, Ruminiclostridium UCG-014*, *Allobaculum*, and *Coprostanoligenes* group were negatively correlated with CRAMP, fatp2, IL-1β, TNF-α, cd36, and fabp2 in ileum of mice (Figure 8A). As for the relationship between gut microbiota and SCFA, we observed a significant negative correlation between acetic acid content and the relative abundance of *Roseburia*. In contrast, *Ruminiclostridium UCG-014* and *Coprostanoligenes group* were positively corrected with acetic acid. In addition, *Ruminiclostridium 1* and *Allobaculum* were positively corrected with acetic acid and propionic acid (Figure 8B).

## 4. Discussion

Our present study demonstrates that administration of *L. plantarum* ZJUIDS14 could significantly alleviate metabolic disorder in mice on a high fat diet. NAFLD is characterized by the accumulation of fat droplets in hepatocytes (22), which is examined by measuring liver weights, liver TG, and histologically with H&E staining of tissue. As expected, *L. plantarum* ZJUIDS14 significantly antagonized liver TG and liver weight increase, suggesting that *L. plantarum* ZJUIDS14 was well able to reduce hepatic steatosis. H&E staining of liver slices exposed liver steatosis and vacuolar degeneration in the HFD group, suggesting that HFD feeding induced histopathological damage. However, treatment with *L. plantarum* ZJUIDS14 could alleviate liver dysfunction and damage, which was further confirmed by promising plasma enzyme levels (ALT and AST levels) and NAS.

Other studies reported that in addition to lipid disorders, NAFLD patients also suffer from insulin resistance (IR) (23), playing a key role in the development of NAFLD by increasing both FFA delivery from adipose tissue and *de novo* lipogenesis in the liver (24). Previous studies have shown that a high-fat diet led to higher OGTT and ITT AUC values (25, 26), which are considered the relevant parameters for scoring IR, glucose tolerance and homeostasis. In agreement with the above, the OGTT and ITT AUC values were significantly higher in the HFD group than in the NFD group. Treatment with *L. plantarum* ZJUIDS14 induced a marked decrease in OGTT and ITT AUC values. Consistent with other studies (27–29), HFD significantly increased the levels of plasma TG, FFA, TC, and LDL-C. Moreover, the plasma levels of HDL-C were increased and TG, FFA, and LDL-C were distinctly reduced after *L. plantarum* ZJUIDS14 treatment when compared with the HFD group. Collectively, *L. plantarum* ZJUIDS14 has been shown to improve dysglycemia and dyslipidemia.

To better understand the mechanism underlying the beneficial effects of *L. plantarum* ZJUIDS14 on lipid metabolism, mRNA levels of genes involved in lipid and fatty acid synthesis were measured. *fatp2*, *fatp5*, and *cd36* are generally responsible for the uptake and transport of fatty acids. Studies revealed that *fatp2* could not only facilitate the import of exogenous FA, but also increase intrinsic acyl-CoA synthase activity to activate FA, which is critical for the production and utilization of lipids (30, 31). *Fatp5* knockout decreased hepatic TG and FFA levels in mice (32). In our study, mRNA expression of *fatp2, fatp5*, and *cd36* in the liver were markedly elevated in the HFD group (*P* < 0.05). In the *L. plantarum* ZJUIDS14 group, higher expression of *fatp2* and *fatp5* was found compared to the HFD group (*P* < 0.05), suggesting that *L. plantarum* ZJUIDS14 facilitated FFA uptake in the liver. In addition, *SREBP-1c* also plays a key role in the induction of hepatic lipogenesis (33). In our study, HFD slightly increased the expression of *SREBP-1c*, which was even higher upregulated due to *L. plantarum* ZJUIDS14 treatment.

Furthermore, we examined the expression of *ppar-α*, which could affect lipolysis by regulating some genes associated with fatty acid β-oxidation, i.e., genes encoding medium-chain acyl-CoA dehydrogenase (*MCAD*) and carnitine palmitoyltransferase 1 (*CPT1*) (34). Drugs targeting PPAR-α has been used in clinical application for NAFLD therapy, since it could promote liver peroxisomal and mitochondrial fatty acid oxidation (35–37). Persistent activation of PPAR-α in ob/ob mice could increase fatty acid oxidation in the liver and reduces obesity (38). Besides, Yoshioka et al. (39) reported that HFD-induced adipocyte hypertrophy and the formation of hepatic lipid droplets were reduced by promoting the expression of ppar-α in liver of mice. In accordance with our results, *ppar-α* mRNA levels were increased in HFD mice (34). However, the level was further up-regulated by treatment with *L. plantarum* ZJUIDS14. Our results were verified at protein levels, suggesting that protein expression of *ppar-α* was further strengthened by *L. plantarum* ZJUIDS14 treatment. Taken together, *L. plantarum* ZJUIDS14 treatment may promote absorption and biosynthesis of fatty acids and triglycerides through increased expression of *fatp2, fatp5*, and *srebp1c*. Secondly, *L. plantarum* ZJUIDS14 treatment significantly upregulated fatty acid β-oxidation and activated lipolysis, which was proved by markedly increased *ppar-α* levels. Taken together, our data indicate that *L. plantarum* ZJUIDS14 alleviates lipid deposition by activation of *ppar-α* in HFD-fed mice.

Once the energy availability in the cell decreased, AMPK was activated, leading to the phosphorylation of its downstream proteins, the inhibition of lipid metabolism and the activation of fatty acid oxidation (40). In our study, AMPK was activated by *L. plantarum* ZJUIDS14 treatment. This was consistent with the increase *ppar-α* in the *L. plantarum* ZJUIDS14 group, indicating that the probiotic protected from hepatic steatosis presumably by increasing fatty acid oxidation and enhancing energy metabolism to some extent. Since AMPK is an activator of mitochondrial respiration (41), *L. plantarum* ZJUIDS14 treatment resulted in activation of AMPK and hence mitigated mitochondrial dysfunction. In our study, HFD decreased levels of DRP1, a marker of stimulated mitochondrial fission. *L. plantarum* ZJUIDS14, however, increased DRP1 levels significantly, suggesting that the probiotic activated mitochondrial fission. In order to examine the function of mitochondrial, OXPHOS activities, relating to insulin sensitivity and resistance to diet-induced obesity (42), were measured. Indeed, *L. plantarum* ZJUIDS14 maintained the homeostatic OXPHOS activities and inhibited their reduction. Thus, *L. plantarum* ZJUIDS14 improved mitochondrial function and ATP production via OXPHOS, which might explain both increases in fatty acid input (synthesis and uptake) and output (export and oxidation).

Hepatic oxidative stress is considered to play an important role in the progression of NAFLD (43). In our present study, liver oxidative stress was assessed by following antioxidant parameters like MDA and T-SOD. MDA is a common product of lipid peroxidation, and MDA level is considered an indicator of oxidative stress (44). SOD protects cells from oxidative stress (45). Our data showed that T-SOD was substantially lower in the HFD group, while MDA contents showed an opposite trend. However, these changes caused by HFD were restored by *L. plantarum* ZJUIDS14 supplementation, suggesting an alleviation of oxidative stress. In order to explore the functional mechanism of the antioxidative actions of *L. plantarum* ZJUIDS14, we examined protein levels related to the Nrf2 signaling pathway, a classic and major regulatory pathway for regulating oxidative stress (46). Nrf2 is an important transcription factor regulating antioxidants against cellular oxidative stress (47). HO-1 exerts anti-oxidative effects by catalyzing heme degradation to produce carbon monoxide (CO) and ferrous ion (48). In our study, administration of *L. plantarum* ZJUIDS14 did not affect the protein levels of Nrf2 and HO-1. Some *PPAR* agonists increase mitochondrial oxidative phosphorylation capacity (49, 50) so we also detected the mitochondria-related protein HO-2. HO-2, present in the mitochondria of hepatocytes, has been shown to improve the extracellular redox state by influencing oxygen consumption and regulating extracellular superoxide dismutase (51). *L. plantarum* ZJUIDS14 indeed increased the HO-2 levels, which was consistent with our results from the OXPHOS activity. Taken together, *L. plantarum* ZJUIDS14 improved antioxidative capacity in HFD-induced NAFLD mice presumably by improving mitochondrial function.

HFD increased the expression of genes involved in fatty acid uptake and transport, including *fatp2*, *fabp2*, and *cd36*, and proinflammatory genes, including *IL-1β* and *TNF-α* in ileum, which were decreased by *L. plantarum* ZJUIDS14 treatment. Thus, *L. plantarum* ZJUIDS14 could modulate lipid metabolism in the ileum tissue and improved immunomodulatory functions. We also examined the mRNA expression level of *cramp*, which is a unique antimicrobial peptide in the gut produced by colonic epithelial cells and playing an important role in regulating gut microbes (52, 53). Mohammad et al. found that *cramp* was significantly higher in patients with T2DM than in healthy subjects and it could regulate lipid accumulation in adipocytes and hepatocytes (54). In our present study, *cramp* was elevated in the HFD group, suggesting the attempt to re-establish the disturbed barrier. However, *L. plantarum* ZJUIDS14 reduced its expression level, indicating that *L. plantarum* ZJUIDS14 could increase the abundance of other probiotics having antibacterial capabilities and leading to a reduced requirement for *cramp*.

Since patients with NAFLD have been found to suffer from gut microbiota dysbiosis (55), probiotics have been proposed as potential candidates to restore gut microbial homeostasis (56). Therefore, in the present study, we examined the effects of *L. plantarum* ZJUIDS14 on improving the metabolic disorder of the intestinal flora. In our study, the PCoA results indicated that *L. plantarum* ZJUIDS14 treatment can indeed modulate gut microbiota in HFD mice. Besides, the relative abundance of bacterial community was restored by ZJUIDS14. Consistent with our findings, Remely et al. reported a decreased bacterial abundance in HFD mice (57). In our study, HFD significantly increased the relative abundance of *Actinobacteria* and decreased that of *Proteobacteria* at the phylum level, in line with the literatures (58, 59). At the genus level, *L. plantarum* ZJUIDS14 administration also prevented changes of *Coprostanoligenes* group, *Ruminococcaceae UCG-014, Allobaculum*, *Ruminiclostridium 1*, and *Roseburia* induced by HFD. *Coprostanoligenes* is a central fecal microbial group for individuals on HFD, mainly through sphingosine to mediate blood lipid metabolism (60). *Ruminococcaceae UCG-014*, *Ruminiclostridium 1*, and *Allobaculum* are SCFA-producing microbiota and play important roles in maintaining a healthy gastrointestinal tract (61–63), improving NASH (64), and alleviating adipose tissue inflammation (65). Consistent with other studies (66, 67), HFD-fed mice had fewer *Ruminococcaceae UCG-014, Ruminiclostridium 1*, and *Allobaculum* than the NFD group, while *L. plantarum* ZJUIDS14 treatment reversed their relative abundance in the colon of HFD-fed mice. Furthermore, *Roseburia* is reported to be a kind of butyrate producer (68). Cai et al. (69) and Yang et al. (70) found HFD increased *Roseburia*, which was consistent with our results. *L. plantarum* ZJUIDS14 intervention decreased *Roseburia* abundance. This may explain the non-significant decrease in butyrate acid induced by HFD. Summarizing, the diversity of the gut microbiome varied little in different groups, but *L. plantarum* ZJUIDS14 increased the relative abundance of SCFA-producing bacteria. Previous studies have placed emphasis on SCFAs in the amelioration of chronic inflammatory diseases, modulation of intestinal barrier function, and promotion of lipid metabolism and obesity (71–73). The levels of SCFAs (acetate, propionate, and butyrate) were decreased to different extents after HFD treatment, consistent with previous studies (74, 75). However, *L. plantarum* ZJUIDS14 increased their levels markedly in the present study, accompanied by the up-regulation of SCFA-producing bacteria. The relationships between gut microbial phylotypes and both ileum parameters as well as SCFA were revealed in Figures 8A, B, respectively, by correlation heatmaps. *Coprostanoligenes* group, *Ruminococcaceae UCG-014*, and *Ruminiclostridium 1* were negatively associated with inflammation and lipid metabolism gene expression in ileum while positively associated with SCFA contents, consistent with the literature (76, 77). In particular, strong negative correlations between *Ruminococcaceae UCG-014* and *CRAMP*, *fabp2*, *fatp2*, and *tnf-α* (ρ = −0.815, ρ = −0.851, ρ = −0.811, and ρ = −0.851, respectively) and postive correlations between *Ruminiclostridium 1* and acetic acid (ρ = 0.726) were observed. Collectively, we concluded that *L. plantarum* ZJUIDS14 was beneficial to the prevention of HFD-induced dysfunctional lipid metabolism, gut dysbiosis, and inflammation in NAFLD.

## 5. Conclusion

In summary, our results revealed that *L. plantarum* ZJUIDS14 treatment significantly mitigated hepatic steatosis and liver damage in HFD mice by modulating gut microbiota balance, intestinal barrier integrity, strengthening mitochondrial function, and increasing fatty acid oxidation. Our new data may pave the way for a novel therapeutic agent, and reinforce the recommendation for the incorporation of LAB into the daily diet, to prevent NAFLD.

## Data availability statement

The original contributions presented in this study are publicly available. This data can be found here: https://www.ncbi.nlm.nih.gov/bioproject/PRJNA891441.

## Ethics statement

The animal study was reviewed and approved by the Institutional Animal Care and Use Committee of Zhejiang Chinese Medical University.

## Author contributions

DR and STL: conceptualization and resources. FC: writing – original draft, data curation, and formal analysis. QD, HZ, SLL, and KC: data curation, formal analysis, and methodology. CL and GY: data curation and investigation. TV: reviewing and editing the manuscript. All authors contributed to the article and approved the submitted version.
