# Supplementary material for: Lactobacillus plantarum ZJUIDS14 alleviates non-alcoholic fatty liver disease in mice in association with modulation in the gut microbiota
Source: Front Nutr. 2023 Jan 9;9:1071284. doi: 10.3389/fnut.2022.1071284 (PMC9868733; doi:10.3389/fnut.2022.1071284)
Supplement: Supplementary file 1 [file Data_Sheet_1.DOCX]

Supplementary Material

## Supplementary Figures


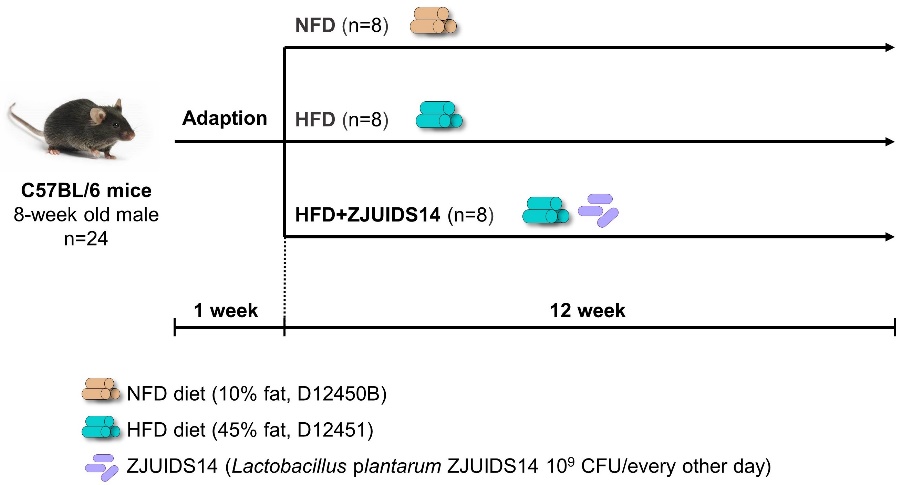


**Figure S1.** **The experimental scheme.**


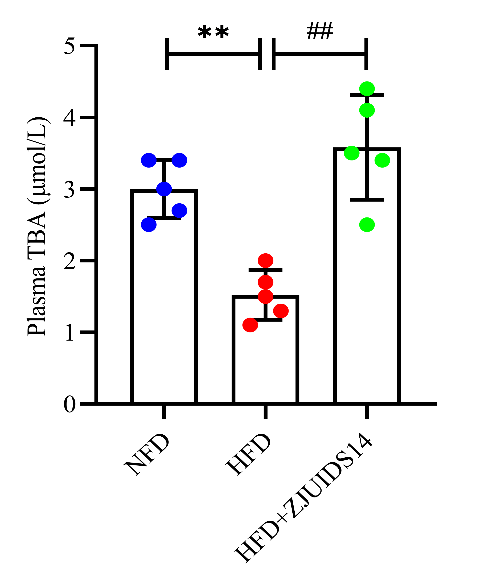


**Figure S2. The effects of *L. plantarum* ZJUIDS14 administration on plasma TBA.** All data are expressed as means ± SD. *^**^P* < 0.01*vs* NFD group; *^##^P* < 0.01 *vs* HFD group.


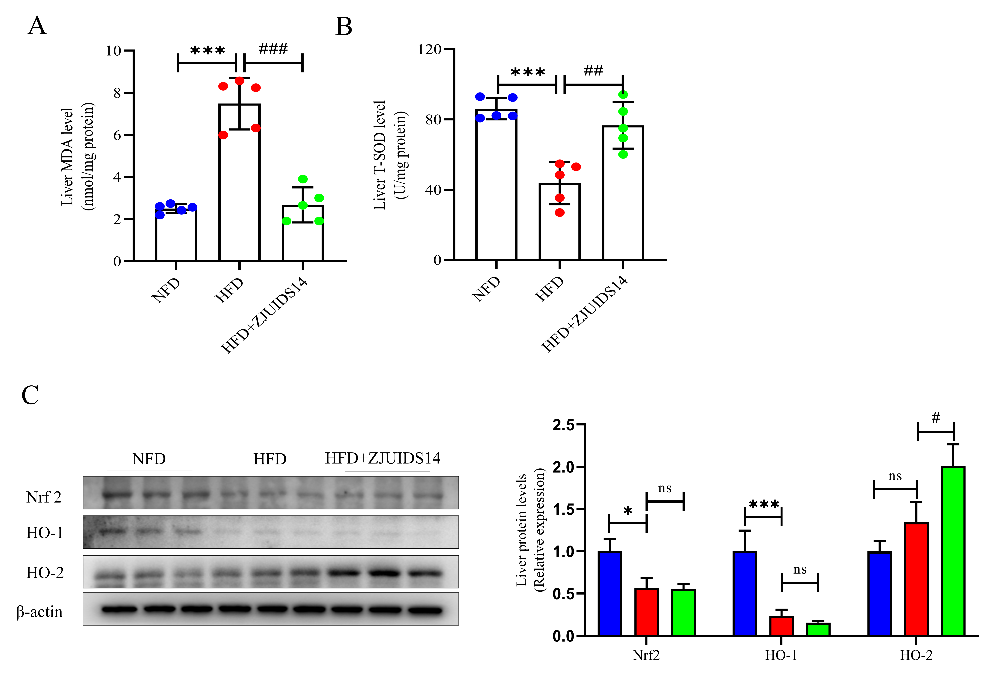


**Figure S3. *L. plantarum* ZJUIDS14 administration improves oxidative stress in HFD fed mice.** (A) Liver MDA level; (B) Liver T-SOD level; (C) Representative immunoblots and protein expression of Nrf2, HO-1, and HO-2 in mice. All data are expressed as means ± SD.*^*^P* < 0.05, *^**^P* < 0.01, ^***^*P* < 0.001 *vs* NFD group; *^#^P* < 0.05, *^##^P* < 0.01, *^###^P* < 0.001 *vs* HFD group.

## Supplementary Tables

Supplementary Table 1. Antibodies for WB.

| Antigen | Dilution | Vendor | Catalog No. |
| --- | --- | --- | --- |
| Nrf2 | 1:1000 | Cell signaling technology | 12721S |
| HO-1 | 1:1000 | Cell signaling technology | [43966](https://www.cellsignal.cn/products/primary-antibodies/ho-1-e3f4s-rabbit-mab/43966?site-search-type=Products&N=4294956287&Ntt=ho-1&fromPage=plp)S |
| HO-2 | 1:2000 | Cell signaling technology | 32790S |
| P-AMPK | 1:1000 | Cell signaling technology | 2535S |
| AMPK | 1:2000 | Cell signaling technology | 2532S |
| OXPHOS | 1:3000 | Abcam | ab110413 |
| ZO-1 | 1:500 | Proteintech Group | 21773-1-AP |
| Claudin-1 | 1:500 | Proteintech Group | 13050-1-AP |
| DRP1 | 1:500 | Proteintech Group | 12957-1-AP |
| OPA1 | 1:500 | Proteintech Group | 27733-1-AP |
| PPARα | 1:1000 | Boster Biological Technology | [A00600-2](https://www.boster.com.cn/home/product/anti-ppara-antibody_a00600-2.html) |
| β-actin | 1:5000 | Boster Biological Technology | [M01263-4](https://www.boster.com.cn/home/product/anti-actin-antibody_m01263-4.html) |
